# An uncommon cause of sub‐acute intestinal obstruction in young adult: Wilkie's syndrome

**DOI:** 10.1002/ccr3.8634

**Published:** 2024-03-26

**Authors:** Kala Shrestha, Niranjan Thapa, Sunil Basukala, Manisha Acharya, Bibek Lama Bhulan, Kaushal Kumar Singh, Oshan Shrestha, Kabita Chaudhary, Milan Kumar Neupane

**Affiliations:** ^1^ Nepalese Army Institute of Health Sciences Kathmandu Nepal; ^2^ Department of Surgery Nepalese Army Institute of Health Sciences Kathmandu Nepal

**Keywords:** acute abdomen, SMA, superior mesenteric artery syndrome, Wilkie syndrome

## Abstract

**Abstract:**

Superior mesenteric artery (SMA) syndrome, also known as Wilkie's syndrome, is a rare disease presenting as an acute abdomen. It has a clinical presentation similar to intestinal obstruction and is often missed during diagnosis. Reduced weight leading to loss of fat pad between SMA and aorta is the main pathophysiology. Diagnosis is made through barium meal and CT scan. Conservative management remains the treatment of choice; however, surgery is opted for in refractory cases.

**Key Clinical Message:**

Superior mesenteric artery (SMA) syndrome, also known as Wilkie's syndrome, is a rare disease presenting as an acute abdomen with clinical features similar to intestinal obstruction. This is a case of SMA syndrome in an adult male with a decrease in aortomesenteric angle, with no predisposing condition.

## Graphical abstract

Summary diagram of anatomical structures involved in Superior mesenteric artery (SMA) syndrome. Left: Healthy patient with regular aorto‐mesenteric angle and distance. Right: apatient with duodenal compression due to SMA syndrome.

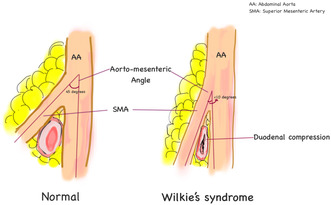

## 1 INTRODUCTION

Superior mesenteric artery (SMA) syndrome, also known as Wilkie syndrome, is a rare disease presenting as an acute abdomen with clinical features similar to intestinal obstruction. Thin lean body habitus and reduced weight leading to loss of fat pad between the SMA and aorta, resulting in a decrease in aorto‐mesenteric angle, is the pathophysiology of this syndrome. The median age of patients is 23 years old and predominant in females over males with a ratio of 3:2. Imaging modalities such as barium meal and CT scan facilitate its diagnosis. The mainstay of management is conservative treatment with decompression using a nasogastric tube, bypassing duodenum with a nasojejunal tube, correction of nutritional deficiencies and electrolyte abnormalities, and total parenteral nutrition or oral nutrition as tolerated. Surgery remains the treatment of choice in refractory cases.^1^

## 2 CASE REPORT

A 31‐year‐old serving soldier with no known comorbidities presented to emergency with complaint of pain abdomen and progressive vomiting for 3 months, increasing in severity since 3 days. Pain abdomen was on and off over the left lumbar and periumbilical region, which was pricking in nature, increasing in severity, with no radiation. It was associated with vomiting which occurred immediately after food consumption, which in particular, worsened for past 3 months. He had multiple episodes of forceful, projectile vomiting containing recently ingested food particles, which was non‐mucoid non‐blood stained and non‐bilious vomiting with associated nausea and shortness of breath. It was relieved to a slighter extent on fasting. He gave history of six to eight episodes of vomiting per day. He also had anorexia and hadn't passed stool for 1 week but had passed flatus. During this period he gave history of significant weight loss of more than 20 kg in the span of last 3 months. His BMI was 18.4 kg/m^2^. There was however no history of fever, loose stool, burning micturition. Bladder function was normal. He had no history of any surgical intervention in the past.

On examination, his general condition was ill‐looking, frail, and wasted. He had a scaphoid abdomen which was soft with no tenderness, no organomegaly, and bowel sounds were present. Other systemic examinations were unremarkable.

### 2.1 Diagnostic assessments

Blood investigations showed complete blood count and random blood sugar levels to be within normal limits. However, blood amylase and lipase were 102 U/L and 122.3μ/L, respectively. Blood urea and creatinine levels were raised with values of 87.7 and 1.42 mg/dL, respectively. Renal function tests showed normal value following fluid resuscitation with a sodium level of 126 mEq/L and a normal potassium level of 4.2 mEq/L.

Ultra sonography showed dilated loops of 5.4 cm, with to and fro motion of intraluminal contents in the right upper and lower quadrant, suggestive of sub‐acute bowel obstruction.

**FIGURE 1:**
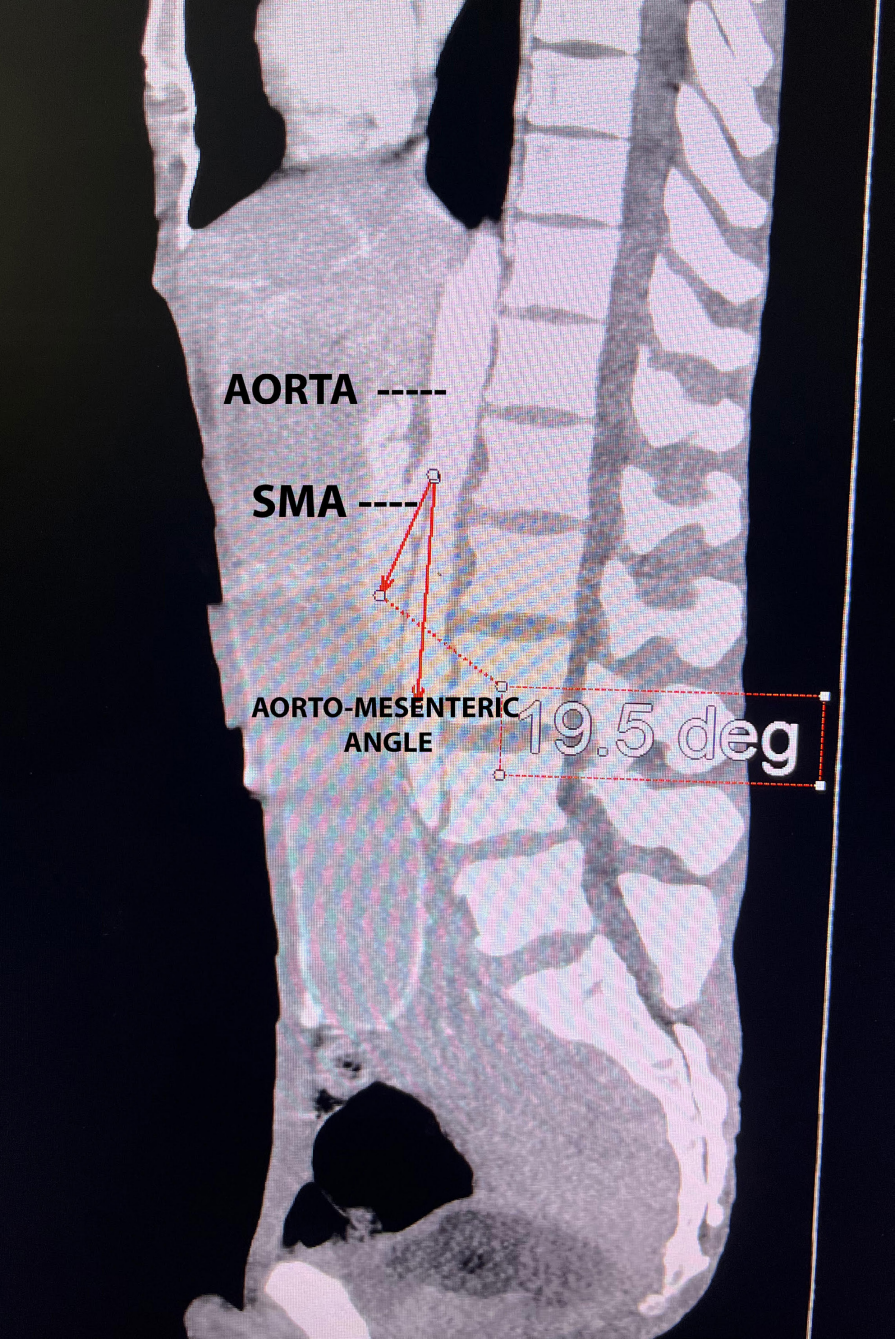
Sagital section of CECT abdomen showing narrowed aorto‐mesenteric angle of 19.5°.

**FIGURE 2:**
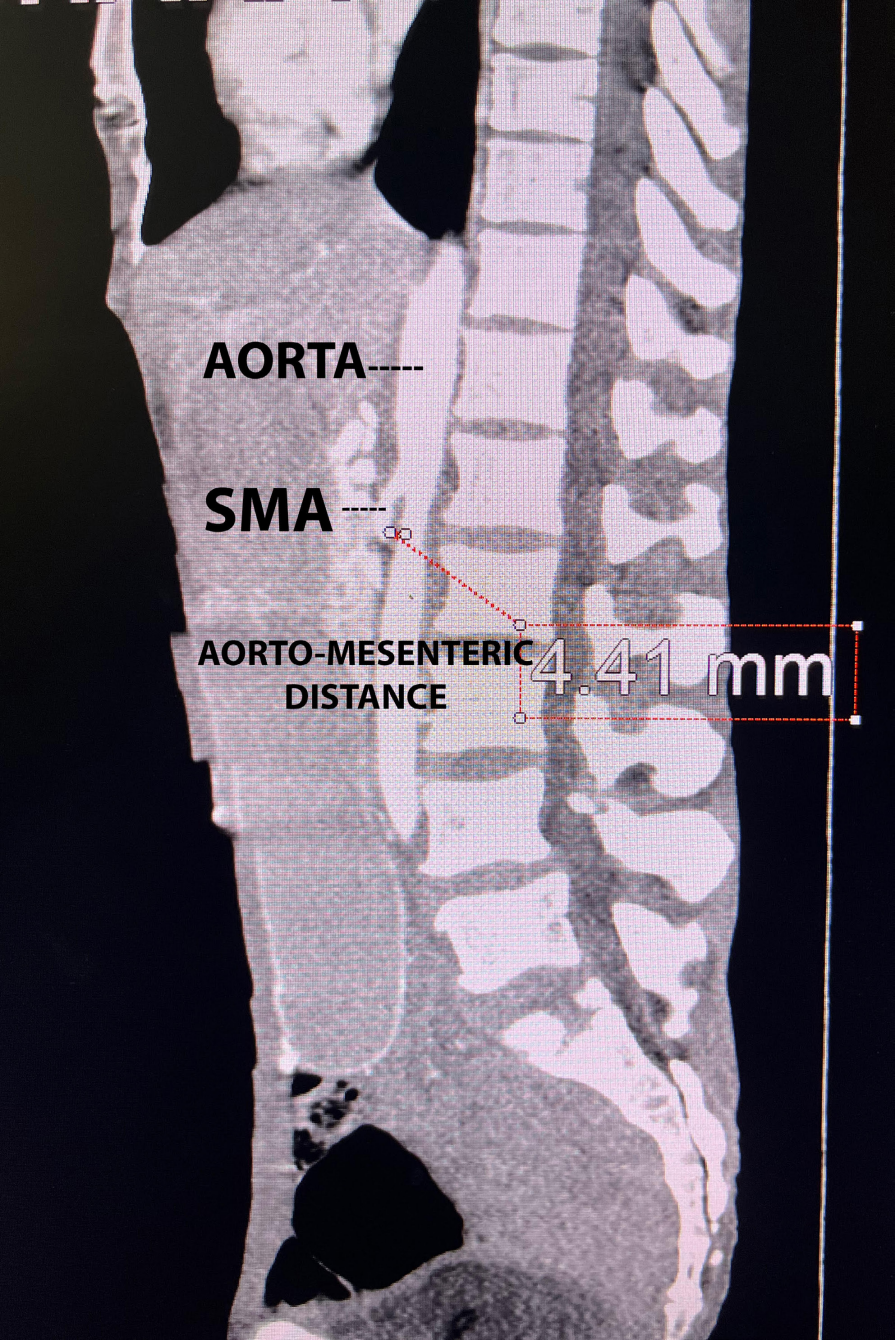
Sagital section of CECT abdomen showing a narrowed aorto‐mesenteric distance of 4.41 mm.

CECT (A+P) showed a grossly distended stomach with abrupt tapering at the junction of the second and third part the duodenum with mass effects. The narrowed aorto‐mesenteric angle (AO) was 19.5° (Figure 1) The decreased aorto‐mesenteric distance was measuring 4.41 mm (Figure 2), all findings concomitant with Wilkie's syndrome.

### 2.2 Treatment

The patient was initially managed in the emergency room, he was kept nil per oral (NPO), and fluid resuscitation along with management of hyponatremia was done by normal saline. Vomiting was managed by ondansetron, along with pantoprazole and the patient was shifted to the ward for further investigation until the confirmation of diagnosis.

After the reports of focused USG and CECT, the diagnosis of SMA syndrome was made. Initial conservative management was attempted with nasogastric decompression, correction of electrolyte imbalances, and nutritional optimization. Later, exploratory laparotomy with retrocolic duodenojejunostomy was done under general anesthesia. In the clinical management of the patient, a conservative approach is favored over surgical intervention. However in our case, due to the patient's progressive symptomatic deterioration, characterized by a substantial weight loss of 20 kg within 3 months, frequent episodes of vomiting, and evident cachexia, and unstable vitals refractory to conservative treatment, the risk–benefit ratio was carefully evaluated. Recognizing the urgency of the situation and the potential benefits of surgical intervention in arresting the patient's declining condition, we made a judicious decision to proceed with surgery rather than continuing with conservative measures. This decision was undertaken with the utmost consideration for the patient's well‐being and in accordance with the best available medical evidence and professional expertise.

Intraoperative findings showed a constriction point in the duodenum which was due to constriction from SMA. He was then kept under observation in the post‐operative ward and kept NPO for 8 h following the surgery and then shifted to the ward after 12 h. He started on a liquid diet after 6 h and tolerated it well. He began regular diet on fourth postoperative day and tolerated it well. He was symptomatically better after the surgery, with improvement in obstructive symptoms but had postoperative pain which resolved gradually. At the time of discharge, he was tolerating full feeds.

### 2.3 Follow‐up

He was discharged with antibiotics (Cefixime and Metronidazole), Paracetamol, and Pantoprazole. The pain resolved after 7 days and he made a full recovery. The patient was advised for alternate day dressing with suture out on the 10th postoperative day. There were no postoperative complications during the follow‐up period. He has been gaining weight and there has been no further complications during his follow‐up period. Patient came to the hospital for follow up on 10th postoperative day for suture removal. During follow up, he was tolerating full feeds. He was advised for follow‐up every 2 months with weight monitoring being done in every visit.

## 3 DISCUSSION

SMA syndrome, also known as Wilkie syndrome, chronic duodenal ileus, arterio‐mesenteric duodenal compression syndrome, and cast syndrome is a rare disease. It is defined as the compression of the third portion of the duodenum between the abdominal aorta and the SMA. Its incidence is estimated to be 0.1%–0.3% and mostly occurs in adolescents and young adults of age 10–39 years. It remains a diagnosis of exclusion and presents with vague symptoms, such as post‐prandial recurrent abdominal pain to more severe abdominal pain, nausea, vomiting, and electrolyte imbalances.^2^ Similar presentation was found in our case with acute abdominal pain and post prandial vomiting. Loss of fat pad between the SMA and aorta due to thin lean body habitus and reduced weight is considered to be the main pathophysiology of SMA syndrome.^3^ The presented case also has a history of weight loss of 20 kg in 3 months and has a thin lean body habitus.

SMA syndrome has been associated with various factors which decrease the acuity of the angle between the aorta and SMA such as malignancy, trauma, substance abuse, burns, spinal or bariatric surgery, and anorexia nervosa.^4^, ^5^

Diagnostic workup requires imaging modalities such as abdominal radiographs, barium studies, and CT findings. Plain abdominal radiographs reveal findings suggestive of small bowel obstruction whereas barium studies show dilation of the first and second part of the duodenum and a relatively collapsed small bowel distal to the point where the SMA crosses the duodenum. In normal CT findings, the aorto‐mesenteric angle and aorto‐mesenteric distance measure 28–650 and 10–34 mm; in SMA syndrome, both parameters are reduced with values to 60–220 and 2–8 mm.^3^ In this case, the aorto‐mesenteric angle was 19.50°, and the aorto‐mesenteric distance was 4.41 mm.

Conservative management with decompression of the stomach using a nasogastric tube, bypassing the duodenum by a nasojejunal tube, correction of nutritional and electrolyte deficiencies by feeding tube or total parenteral nutrition remains the mainstay of management. The goal is to improve the nutritional status which in turn builds up the fat cushion between SMA and the aorta. Prone position, left lateral decubitus, or knee‐chest position has been found to relieve symptoms by removing tension from the mesentery and the SMA, and opening the space between SMA and aorta.^6^ Use of enteral nutrition with high calorie diet has been found to be effective in early recovery from symptoms and increase in weight gain.^7^ Medical treatment may be successful in patients with a short history, moderate symptoms and incomplete duodenal obstruction.^8^ Surgical management has been proven to be effective in cases with severe symptoms and failure of conservative management with duodenojejunostomy, being the preferred surgical approach.^3^, ^9^

SMA syndrome is thus, a rare disease resulting in life‐threatening outcomes. A high index of suspicion is a must in cases of acute abdomen especially when it is associated with post‐prandial pain and weight loss.

## 4 CONCLUSION

The case of Wilkie syndrome is rare and can have a non‐specific clinical presentation. Contrast enhanced CT remains the gold standard diagnostic modality. Use of enteral nutrition has been reported as an effective option for relief of symptoms leading to an increase in weight gain. In severe cases or cases refractory to conservative management, surgical management with duodenojejunostomy is preferred.

## AUTHOR CONTRIBUTIONS

**Kala Shrestha:** Conceptualization; writing – original draft. **Milan Kumar Neupane:** Writing – review and editing. **Kabita Chaudhary:** Writing – review and editing. **Oshan Shrestha:** Writing – review and editing. **Sunil Basukala:** Conceptualization. **Niranjan Thapa:** Writing – review and editing. **Manisha Acharya:** Writing – original draft. **Kaushal Kumar Singh:** Writing – review and editing. **Bibek Lama Bhulan:** Writing – original draft.

## FUNDING INFORMATION

This article did not receive any grants.

## CONFLICT OF INTEREST STATEMENT

The authors have no conflict of interest to declare.

## CONSENT

Written informed consent was obtained from the patient to publish this report in accordance with the journal's patient consent policy.

## Data Availability

All the findings are present within the manuscript.
